# Using Evidence-Based Medicine to Support Clinical Decision-Making in RMS

**DOI:** 10.3390/cancers15010066

**Published:** 2022-12-22

**Authors:** Robert S. Phillips, Bas Vaarwerk, Jessica E. Morgan

**Affiliations:** 1 Centre for Reviews and Dissemination, University of York, York YO10 5DD, UK; 2 Department of Paediatric Haematology and Oncology, Leeds Children’s Hospital, Leeds LS1 3EX, UK; 3 Department of Paediatrics, Amsterdam UMC—Emma Children’s Hospital, University of Amsterdam, 1105 AZ Amsterdam, The Netherlands

**Keywords:** evidence-based medicine, rhabdomyosarcoma, patient and public involvement

## Abstract

**Simple Summary:**

Evidence-based medicine uses healthcare professional experience, research evidence, and patient preferences to support the best decisions in healthcare. Within this manuscript we outline the different ways that research methods and infrastructure have been developed to help support evidence-based decision-making in rhabdomyosarcoma. We discuss key issues in various areas including involving families in the research process, selecting aspects of the research design (topics and outcomes), drawing research together (evidence synthesis and guideline development), sharing research findings, and supporting research processes (including research about research).

**Abstract:**

The foundations of evidence-based practice are the triad of patient values and preferences, healthcare professional experience, and best available evidence, used together to inform clinical decision-making. Within the field of rhabdomyosarcoma, collaborative groups such as the European Paediatric Soft Tissue Sarcoma Group (EpSSG) have worked to develop evidence to support this process. We have explored many of the key research developments within this review, including patient and public involvement, decision-making research, research into areas other than drug development, core outcome sets, reporting and dissemination of research, evidence synthesis, guideline development and clinical decision rules, research of research methodologies, and supporting research in RMS.

## 1. What Is Evidence-Based Medicine?

Evidence-based medicine (EBM) refers to bringing together researchers, patients, and clinicians, through integration of best available evidence, with clinical experience and patient priorities or preferences, to support clinical decision-making. EBM has become well established in many areas of paediatric oncology, including the care of children and young people with rhabdomyosarcoma (RMS), where well-designed preclinical and clinical work have guided improvements in survival and patient experience. Originally, EBM teaching referred to a single hierarchical evidence pyramid, which aimed to help define “best available evidence” [1]. Over time, recognition of the nuances of research methods, and the best ways to answer different clinical questions means that the roles and values of different methodologies have developed, but holding to the core idea of research best placed to reflect the truth. 

One of the fundamental tenets of research is that collaborative working improves outcomes. To achieve this, research networks have developed, usually geographically based and content-specific, including the European Paediatric Soft Tissue Sarcoma Group (EpSSG). This has facilitated the conception, design, and delivery of large, often multinational studies to address essential questions in the care of children with rhabdomyosarcoma. Many of these successes have been described elsewhere within this Special Issue. To date, collaborations have particularly focused on describing clinical experiences and outcomes, early phase studies, and systemic therapy trials. These provide many well-established research processes, which can now be used to move other areas of care and treatment forward.

This manuscript aims to address some new and emerging research developments, as well as areas of discussion and debate in research to best support the care of children and young people with RMS. We also cover some of the issues around implementation of research, and how best to support research infrastructure, so that findings are more easily transferred from “bench to bedside”, drawing on our own experiences as well as the work of the various collaborative groups in RMS research.

## 2. Patient and Parent Involvement

Working in partnership with patients and their families when developing, undertaking, and disseminating the findings of research projects is now widely recognised as an ethical imperative and a way of making research more effective and efficient. Many different approaches have been used in RMS, and patient/public involvement (PPI) has been integrated into the frontline and relapsed (FaR)-RMS and REFoRMS studies.

**Figure 1:**
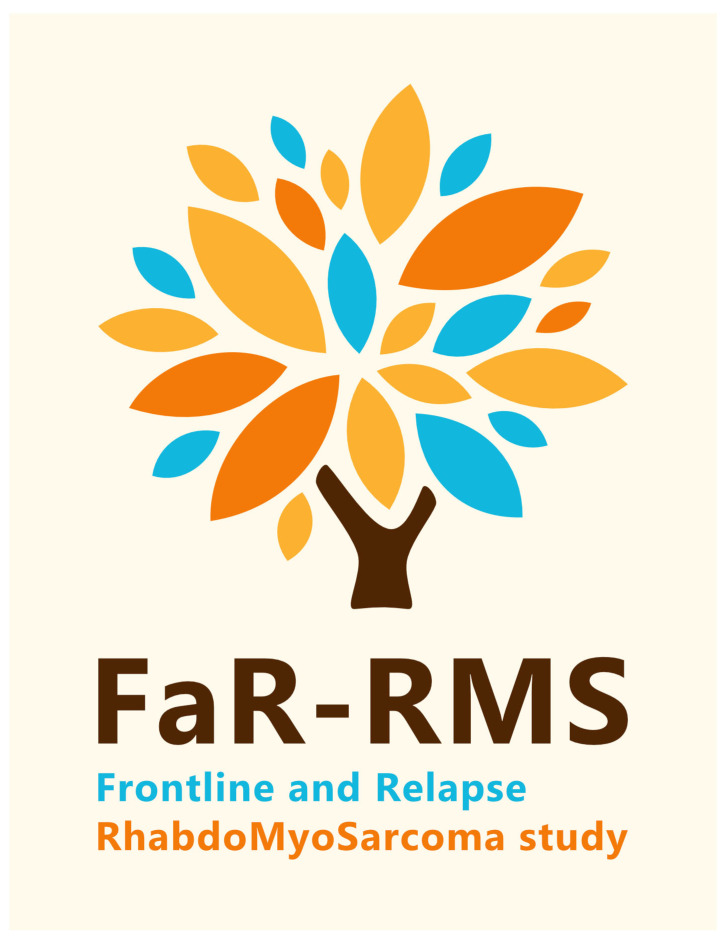
FaR-RMS logo developed in collaboration with young cancer survivors (kindly supplied by Mrs S Wakeling).

Before a study hits the desk of the departmental head, funder, or ethics committee, PPI may have shaped it. Joint priority in setting partnerships, drawing input from patients, clinicians, and researchers have been used to outline the key areas of research interest and importance [2,3,4]. PPI should shape the nature of the question being asked, with shared responsibility for the direction of researcher. This germ of a study then grows, nurtured by all the stakeholders, and continues to engage PPI. This might well need specific training in some of the technical aspects of studies and their governance, but this investment pays off. PPI can make studies run more practically—those who have been through the experience of being treated have a better idea of burden and logistics than those of us by benches and desks. PPI can massively improve the communication materials we use, as demonstrated by the FaR-RMS logo, which was developed alongside young cancer survivors (see Figure 1). Making our study results understandable by families and patients often means we obtain better understanding and wider knowledge from our clinical colleagues too. 

The REFoRMS project was born from a desire by patient families to know more, and know better, what early phase trials had shown previously. The protocol was honed by a group of families with experience of relapsed or refractory RMS, and key elements of data to be collected and methods of analysis were modified by the PPI group. Within evidence synthesis projects, particularly where no core outcome set exists, PPI involvement can powerfully demonstrate where primary researchers have not collected data, which is of critical value to those participating in the research. The next phase of this study will include a study-within-a-study generated by the PPI group, and investigations into the best ways of communicating outcomes, including adverse events/side effects.

## 3. Decision-Making Research

Within the practice of evidence-based medicine, sharing decision-making between clinicians and patients is considered particularly important. In paediatric practice, this also includes sharing decisions with children and young people, commensurate with their development. This process is particularly important where there is no clear recommended best option, for example, in RMS in the setting of multiply relapsed or refractory disease. Research around decision making in poor prognosis children’s cancer was recently synthesised in a scoping review, which described parents’ values and preferences when making decisions, explored the role of various factors influencing decision-making, and stressed the importance of involving families in choices about care [5]. Decision-making specifically in relapsed and refractory RMS is currently being studied within a case study-based qualitative study within the REFoRMS project, with results anticipated in 2023 [6]. Through understanding how treatment-related decisions are made by families, researchers can help healthcare professionals understand and support this process in clinical practice in order to help reduce future decisional regret.

## 4. Research into Aspects other Than Drug Development

In addition to the studies focusing on the optimal therapeutic treatment for patients with RMS, the collaboration within the international cooperative groups has also generated research interest in other aspects such as imaging, supportive care, and quality of life. 

Imaging plays a pivotal role in staging, response assessment, and during follow-up of patients with RMS. A joint effort between collaborative groups has recently led to the development of a European guideline for imaging in RMS [7]. This guideline not only provides recommendations on appropriate modalities and technical protocols, but it also provides standardized reporting templates. Adherence to these guidelines will lead to international harmonisation to ensure optimal imaging for all patients and will also facilitates imaging research. Ideally, central radiology review is part of future trials, to improve reporting harmonisation and to improve the quality of these studies. The QUARTET (Quality and Excellence in Radiotherapy and Imaging for Children and Adolescents with Cancer across Europe in Clinical Trials) platform, initiated to enable centralized quality assurance of radiotherapy to improve quality of treatment, also enables central collection and central review of imaging data and this will further expand our knowledge on imaging in RMS [8]. 

In recent years, the attention of researchers has shifted from objective outcome measures solely to an increasing focus on patient-reported outcome measures (PROM). The most frequently used PROM is health-related quality of life (HRQoL). Studies in RMS focus on improving survival for patients; however, HRQoL measurement by PROMs could play an important part in determining the burden of treatment, especially in cases where no difference in survival was found between different treatment regimens. So far, only a few studies have evaluated HRQoL in patients treated for RMS. These studies have focused on specific patients and/or treatment characteristics, for example specific local therapy regimens [9,10,11]. In current RMS trials, HRQoL is frequently used as a secondary outcome measure to determine the efficacy of treatment. Furthermore, HRQoL can be incorporated into economic analyses of different interventions and thus guide cost-effective health service design.

## 5. Core Outcome Sets 

One of the challenges in RMS research is that many studies use different outcomes. While this is justifiable in some settings, for example the absence of estimates of diagnostic accuracy in a study of extended-duration maintenance therapy, for studies with a similar function there should be a key set of objectives that are central to the research and reported to inform patients, clinicians and other researchers. This problem is more obvious when undertaking a comparison of outcomes in evidence synthesis by meta-analyses. For instance, multiple studies have reported on the value of early tumour size response assessment and its impact on survival, however these studies used different response criteria, making it difficult to perform meta-analyses on the data [12].

The development of core outcome sets could overcome the problem of heterogeneity by providing a minimal set of outcomes to be described within clinical trials, which can be supplemented with further study-specific items [13]. Furthermore, this could overcome problems with selective reporting and increase the relevance trial reports. A core outcome set is a consensus-based standardized set of outcomes that should be measured and reported in all trials within the field. This set could be developed with different stakeholders within the area of research, such as physicians, patients, and parents, increasing the involvement in research of the last two groups. The Core Outcome Measures in Effectiveness Trials (COMET) initiative maintains a database of core outcomes sets; so far, no specific set for RMS is registered. We strongly encouraged the development of multiple core outcome sets for different type of studies in RMS. 

## 6. Reporting and Dissemination of Research

Effective reporting and disseminating of research findings is essential for optimising the utility and impact of the data gathered, and a number of initiatives have been developed to improve this aspect of the research process. The AllTrials campaign calls for the reporting of every clinical trial, so that no collected information is lost and the risk of bias within available evidence is reduced [14]. This concept is supported by ethical research principles, and reduces research waste, both in terms of funding and time. The establishment of clinical trials registries such as ClinicalTrials.gov facilitates this reporting of clinical trial results, whilst also allowing researchers to reduce duplication of studies that are currently ongoing. 

The dissemination (or sharing) of research results can take many different forms. Traditionally, researchers write scholarly articles for publication in scientific journals. Processes that have been developed to improve this form of dissemination include reporting guidelines such as the CONSORT statement for clinical trials or the PRISMA statement for systematic reviews [15,16]. These frameworks, which are now required by many journal publishers, guide researchers to consistently provide the essential details of their methods and findings so that readers are best able to understand the strengths and weaknesses of the work.

Additional work in the development of pooled anonymous data sets for secondary analyses has progressed in other areas of research, such as the Yale University Open Data Access (YODA) project [17]. Within paediatric oncology, more specific data collaboratives such as the PICNICC group in febrile neutropenia or MAGIC in germ cell tumour have allowed a far more nuanced understanding of risk stratification [18,19].

Beyond traditional publishing and sharing research through posters or presentations at academic conferences, researchers and funders of research have worked to share their findings through a variety of means with broader audiences, including with patients and parents. For example, the UK’s Children’s Cancer and Leukaemia Group (CCLG) host an annual research conference for patients and parents, have a regular patient and parent-focused online research webinar, use social media sites such as Facebook and Twitter to share information about ongoing research, and have a magazine “Contact” specifically designed for families with experience of cancer. In each of these for a, research relating to rhabdomyosarcoma has been shared with the wider community. 

## 7. Evidence Synthesis

Since the 1990s, it has been recognised that systematic searching, appraisal, and synthesis of available evidence in a research area can provide more accurate estimates of outcomes, facilitate the exploration of subgroup differences, and identify gaps in the evidence base for further investigation. This has been well-used in RMS, including diagnostic, therapeutic, and prognostic issues. For example, a review of the use of functional imaging demonstrated the increased initial disease detection rates of PET/PET-CT and recommended further studies of PET-CT as a prognostic tool in RMS [20]. This has informed the design of the EpSSG FaR-RMS protocol, which includes a secondary objective to address this clinical question. 

Whilst initial systematic reviews focused on the synthesis of data from randomised clinical trials, methodologies have developed to support different clinical questions. Qualitative syntheses use interview, focus group, and ethnographic data including that from complex interventions. Network meta-analyses (NMAs) allow more detailed assessments of interventions where direct comparison data makes it difficult to draw conclusions. Meanwhile, individual patient data (IPD) meta-analyses draw together participant-level data rather than aggregate summaries of studies. 

One particular novel approach being used in RMS research is that of living systematic reviews, where regular or continuous surveillance of evidence feeds to into syntheses that are updated to reflect the most recent research in a specific field. They have most famously been used to track evidence emerging related to the COVID-19 pandemic. Within RMS, the Living-REFoRMS project is currently under development as the first living systematic review in children’s cancer. It will provide an interactive online resource of the data from a living systematic review of early phase studies for children and young people with relapsed and refractory RMS, including both completed and currently ongoing studies [21].

## 8. Guideline Development and Clinical Decision Rules

The development of a high-quality clinical practice guideline (CPG) is a positive step towards integrating the best clinical evidence and patient-important outcomes to guide expert healthcare professionals in their care of individual children and their families. CPGs should mirror the evidence-based healthcare paradigm and be transparent, explicit, and nuanced.

In the setting of RMS, CPGs are necessary to bridge the gap between clinical trial protocols and what we do in the wards and clinics. A CPG will often mirror a trial protocol in the way it stratifies risk, undertakes diagnostic, safety and response evaluations, and dose therapies. Where it should differ is in the mandating of extra investigations that are necessary for regulatory authorities, or assessment points that may be informative in a trial but have no bearing on management, or in follow-up scheduling where the objectives of everyday work differ from the need to have a homogeneous dataset to answer trial questions. 

The production of a CPG is a step towards everyday use, but they frequently require local thinking and modification in some way. Occasionally, a CPG can simply be adopted—put directly into practice. Mostly, it will need contextualising—training in specific areas or adding localised information or processes. Sometimes, a formal adaptation is essential—changing recommendations on the basis of local variations of drug licencing, resource, or acceptability [22].

## 9. Research of Research Methodologies

Improving the methods for performing research increases the quality of the findings and makes research more efficient in terms of time and resources used. Within rhabdomyosarcoma, novel research methodologies are being introduced in various ways. One clear example is the FaR-RMS study, which uses a platform approach to address multiple key clinical questions in the most efficient way [23]. Furthermore, the study uses a multi-arm, multi-stage (MAMS) approach to evaluate different potential treatments for both frontline and relapsed disease. MAMS designs allow for responsive research with reduced regulatory, set-up, and administrative costs [24]. They also accelerate understanding of the best treatments for a condition by reducing the need for sequential studies of different interventions.

Another area where research methodologies continue to develop is in that of local therapies studies. Traditionally, these have been more challenging to evaluate as they are not well conceptualized within the phases of research developed for systemic therapies. However, progress has been made with radiotherapy randomisations now included within the FaR-RMS study, including dose-finding, timing, and localization questions [23]. Furthermore, structured frameworks have been developed for the evaluation of surgical interventions, and there are now well-established programmes of combined local therapies, such as the AMORE approach [25,26,27].

## 10. Supporting Research in RMS

Demonstrated throughout this Special Issue, research plays a key role in understanding RMS and developing interventions to improve the care of children and young people who develop the disease. Mechanisms to support research and the researchers involved are key to improving the quality and efficiency of RMS care. Researcher development includes training and education programmes at local, regional and international levels, including broader initiatives such as Young SIOP and Young SIOP-E, which aim to build networks and opportunities for junior paediatric oncologists. Protecting research time, high-quality mentorship, and supportive institutional and broader infrastructure is known to support clinical academic careers, and thus funding these areas alongside specific research costs is a key priority for supporting childhood cancer research [28].

Research infrastructure, which aims to speed up processes or improve access to data and/or techniques, has also been a key area of recent development. One such area is the International Soft Tissue SaRcoma ConsorTium (INSTruCT), which provides a data commons, where de-identified data from clinical trials of RMS performed by collaborative groups across the world are pooled, using a common data dictionary, and available to researchers on request for further analyses [29]. The INSTruCT dataset optimises the use of the data collected and provides a larger pool of information from which to draw conclusions about various clinical questions. This has already led to multiple outputs and publications, beyond those described in the contributing original research. 

Meanwhile, the ACCELERATE initiative was established as an international organisation to improve new drug development for children with cancer, with impacts on aspects of research design, policy, and stakeholder engagement [30]. Key activities have included forums to prioritise drugs for development, working groups to address particular challenges, educational and networking events to improve communication between stakeholders, and advocacy that has impacted key policies around novel agent research in children and adolescents.

As demonstrated within this manuscript, and indeed evident throughout this Special Issue, there has been a large amount of varied and heterogeneous research on RMS. Combining individual studies can be achieved through evidence synthesis as we have already discussed, but overviews of the whole landscape of RMS research are best achieved through collaborative international groups such as the EpSSG. These groups bring together leading experts in the field thus facilitating the harmonization of research practices and preventing unnecessary duplication of work. Furthermore, expert committees help to identify new contributions, situating these within the wider context, and supporting their integration into existing programmes of work. 

## 11. Summary

In summary, there have been extensive developments in the design and delivery of research for children and young people with rhabdomyosarcoma. This has resulted in improved outcomes in terms of both the quantity and quality of survival. Nonetheless, there are multiple opportunities for further improvements in research practice and process to continue to build upon these firm foundations, for which the infrastructure and support is now clearly established. Collaborative groups such as the EpSSG will be essential stakeholders in future endeavours.

## Data Availability

Not applicable.
